# Pleasant Pain Relief and Inhibitory Conditioned Pain Modulation: A Psychophysical Study

**DOI:** 10.1155/2018/1935056

**Published:** 2018-06-03

**Authors:** Nathalie Bitar, Serge Marchand, Stéphane Potvin

**Affiliations:** ^1^Centre de recherche de l'Institut Universitaire en Santé Mentale de Montréal, Montreal, QC, Canada; ^2^Department of Psychiatry, Faculty of Medicine, Université de Montréal, Montréal, QC, Canada; ^3^Centre de recherche du Centre Hospitalier de l'Université de Sherbrooke, Sherbrooke, QC, Canada; ^4^Department of Surgery, Faculty of Medicine and Health Sciences, Université de Sherbrooke, Sherbrooke, QC, Canada

## Abstract

**Background:**

Inhibitory conditioned pain modulation (ICPM) is one of the principal endogenous pain inhibition mechanisms and is triggered by strong nociceptive stimuli. Recently, it has been shown that feelings of pleasantness are experienced after the interruption of noxious stimuli. Given that pleasant stimuli have analgesic effects, it is therefore possible that the ICPM effect is explained by the confounding effect of pleasant pain relief. The current study sought to verify this assumption.

**Methods:**

Twenty-seven healthy volunteers were recruited. Thermal pain thresholds were measured using a Peltier thermode. ICPM was then measured by administering a tonic thermal stimulus before and after a cold-pressor test (CPT). Following the readministration of the CPT, pleasant pain relief was measured for 4 minutes. According to the opponent process theory, pleasant relief should be elicited following the interruption of a noxious stimulus.

**Results:**

The interruption of the CPT induced a *mean* and *peak* pleasant pain relief of almost 40% and 70%, respectively. Pleasant pain relief did not correlate with ICPM amplitude but was positively correlated with pain level during the CPT. Finally, a negative correlation was observed between pleasant pain relief and anxiety.

**Discussion:**

Results show that the cessation of a strong nociceptive stimulus elicits potent pleasant pain relief. The lack of correlation between ICPM and pleasant pain relief suggests that the ICPM effect, as measured by sequential paradigms, is unlikely to be fully explained by a pleasant pain relief phenomenon.

## 1. Introduction

Chronic pain affects approximately 22% of the adult population [1] and is a complex phenomenon resulting from biological, psychological, and social factors. Among these factors, the importance of central mechanisms, such as the activity of endogenous pain excitatory and inhibitory systems, is increasingly acknowledged [2–4]. Indeed, growing evidence suggests that endogenous pain modulation mechanisms are impaired in nearly every type of chronic pain disorders, and that alterations are particularly significant in neuropathic and functional pain syndromes [5–7].

*Inhibitory conditioned pain modulation* (ICPM) is one of the principal endogenous pain inhibition mechanisms [8–10]. The ICPM theory postulates that a nociceptive stimulation will reduce another nociceptive stimulation if it occurs on a body surface distant from the pain surface [11, 12]. Preclinical studies have shown that the ICPM effect is mediated by brain stem and bulbospinal mechanisms [13–16]. When triggered, ICPM causes a diffuse diminution of pain throughout the body.

From an experimental point of view, two types of paradigms are used to measure ICPM: in the *parallel* ICPM paradigm, a noxious stimulus (test stimulus) is applied before and at the same time as a heterotopic conditioning painful stimulus, while in the *sequential* paradigm, the test stimulus is applied before and after a heterotopic conditioning painful stimulus [17]. Considering that it is unclear if the *parallel* ICPM paradigm truly measures the ICPM effect or a distracting effect, some investigators prefer the *sequential* paradigm which removes the potential effect of distraction [18–20]. It is indeed well known that pain experience is reduced when individuals are engaged in cognitive tasks (e.g., arithmetic and working memory) [10]. This raises the possibility that the conditioning stimulus actually distracts participants from their pain when it is concomitantly administered at the same time as the test stimulus. Conversely, some laboratories have made mention of their preference of the *parallel* ICPM paradigm over the *sequential* one, considering that ICPM effect gradually fades over time and that the precise duration of this effect remains uncertain [21].

Another potential limitation of *sequential* ICPM paradigms that has gone unnoticed is that the pain reduction observed using these paradigms may be confounded by the pleasant pain relief phenomenon. According to the opponent process theory, when a stimulus causing deviation from homeostasis is terminated, the opposite sensation will be felt [22]. Consistently with this theory, recent research has shown that the interruption of a noxious stimulus causes a feeling of pleasantness [23], similar to the feeling often observed in reaction to analgesic drugs [23]. Given that pleasant stimuli (e.g., music, odors, and attractive faces) are well known for producing analgesic effects [24–26], it is therefore possible that the interruption of the conditioning stimulus elicits a pleasant feeling, which in turn decreases pain perception when the second test stimulus is reapplied. If so, the reduction in pain perception observed during the second test stimulus would not reflect a pure ICPM effect but rather a pleasure-induced analgesic effect, at least partially.

In the past, our research team has pursued several studies on ICPM using a sequential paradigm, consisting in the application of a tonic noxious heat stimulation to the left forearm of participants eliciting moderate pain, administered before and after the immersion of their right arm in a bath of cold water. This paradigm has allowed us, among others, to show that pain perception is reduced during the second application of the test stimulus, relative to the first one, indicating that endogenous pain inhibition mechanisms have been recruited [27, 28]. In the current study, we sought to examine a hypothetical association between ICPM and pleasant pain relief, using our validated ICPM procedure [3]. Thus far, most studies on pleasant pain relief have used heating thermodes to elicit the phenomenon [23, 29]. The current study differed from the latter, and we measured pleasant pain relief after the interruption of the cold-pressor test, given that it is the conditioning stimulus used in our *sequential* paradigm to trigger the ICPM effect. The secondary objective of the current study was to examine the potential associations between pleasant pain relief and anxiodepressive subclinical symptoms. Although several experimental studies have shown that anxiety and depression influence pain perception in experimental settings [30–32], the influence of these variables on pleasant pain relief is unknown.

## 2. Method

### 2.1. Participants

We recruited a total of 27 (14 women) healthy participants, aged between 18 and 35 years old (mean age 25.1 years ± 4.27, mean ± standard error of the mean (SEM)) (Table 1). Exclusion criteria were the following: (1) any DSM-V axis psychiatric disorder (including substance use disorders); (2) centrally acting medications; (3) neurologic disorders; and (4) any unstable medical condition. In particular, none of the participants suffered from chronic pain and none had significant acute painful symptoms as determined with the *Brief Pain Inventory* (mean pain = 0.9 ± 0.4) [33, 34]. Subclinical psychological symptoms (e.g., depression, anxiety, anhedonia, and pain) were evaluated, respectively, with the French versions of the *Beck Depression Inventory-II* (BDI-II) [35], the *State and Trait Anxiety Inventory*-state subscale (STAI-S) [36, 37], and the *Snaith–Hamilton Pleasure Scale* (SHPS) [38, 39]. Recruitment was made via word of mouth and through online advertisement (Kijiji). Each participant signed a detailed consent form, and the local ethics committee approved the research.

### 2.2. Inhibitory Conditioned Pain Modulation (ICPM) Paradigm

#### 2.2.1. Heat Pain Threshold and Tolerance

Thermal pain threshold and tolerance were measured by applying a 3 cm^2^ Peltier thermode (TSA II, Medoc, Advanced Medical Systems, Ramat Yishai, Israel) on the left forearm of participants [28]. This heating plate was connected to a computer and allowed a precise control of temperatures. Experimental temperatures were initially set at 32°C and gradually increased at a rate of 0.3°C per second. To ensure that there would be no peripheral sensitization, the thermode was moved to a different area of the forearm for every test. Participants were asked to report the moment at which sensation changed from heat to pain (thermal pain threshold, VAS = 1) [23, 28] and the moment the sensation of pain was at its highest (most intense pain tolerable) (thermal pain tolerance, VAS = 100). For each participant, the temperature inducing moderate pain (T50) was also measured. Upon the first application, these measures were taken verbally to ensure the participant's comprehension of the procedure. During the second and third applications, these measures were reported by the participants using a computerized visual analog scale (VAS). This scale ranged from 0 (no pain) to 100 (most intense pain tolerable) [28].

#### 2.2.2. Tonic Heat Pain Perception

The *test stimulus* consisted of a continuous heat stimulation that induced moderate pain (T50) for 2 minutes [28]. This heat stimulation was administered with a thermode on the left forearm of the participants. The temperature of the thermode quickly reached T50, an individually predetermined temperature (baseline at 32°C and increase rate of 0.3°C per second), and then remained constant for the remaining time. However, participants were not told that the temperature was kept constant [40]. During the administration of the test stimulus, individuals were instructed to measure pain intensity using the same COVAS as previously mentioned. The test stimulus was administered twice, separated by the administration of the cold-pressor test (CPT) (e.g., the conditioning stimulus).

#### 2.2.3. Conditioning Stimulus

The CPT consisted the immersion of the opposite arm (right arm) into a bath of ice water that was kept constant at 10°C, for a maximum of 2 minutes, by continuously recirculating the water (Julabo F33-HL heating/refrigerated circulator). The temperature was chosen to be painful enough to elicit the endogenous analgesic effect yet tolerable for 2 minutes [28]. During the administration of the conditioning stimulus, participants were instructed to verbally report pain intensity and pain unpleasantness on a scale of 0 to 100. In order to differentiate between pain intensity and pain unpleasantness, two scenarios were presented to the participants. For pain intensity, they were asked to imagine themselves at their favourite concert; the music is extremely loud and it damages their eardrums. In this scenario, the intensity is very high; however, it is not unpleasant because they enjoy the music. On the contrary, for pain unpleasantness, they were asked to imagine themselves studying the day before a final exam with loud construction noise outside their house. In the second scenario, the intensity of the noise is not high; however, it is extremely unpleasant. The measures for pain intensity and pain unpleasantness were taken at the moment the arm was immersed into the bath of cold water and afterwards every 30 seconds, until 120 seconds. With these measures, the mean pain intensity and mean pain unpleasantness were calculated for each participant. By measuring pain perception (using the test stimulus) before and after the conditioning stimulus, it was possible to measure ICPM. In other words, ICPM is defined as the reduction in pain perception observed between both administrations of the test stimulus (before and after the conditioning stimulus) [20].

#### 2.2.4. Pleasant Pain Relief

Pleasant pain relief was measured immediately after the conditioning stimulus. In order to explain to participants the pleasant pain relief phenomenon, we provided an example similar to the one used by Leknes et al. [23]. Participants were asked to imagine themselves walking in a −30°C snowstorm for 20 minutes and finally arriving home to feel the warmth of the air inside the house. This warmth would induce the feeling of both pain relief and of pleasure [23]. Considering that the ICPM effect lasts for a short time span (approximately 10 minutes), it was important that the administration of the second test stimulus quickly follows the conditioning stimulus [5]. Consequently, following the conditioning stimulus, the measure of pleasant pain relief was taken only once in order to avoid delaying the administration of the second test stimulus. The second test stimulus was then administered immediately after the score of pleasant pain relief was taken. To fully capture the dynamics of pleasant pain relief, thirty minutes after the full administration of the sequential ICPM paradigm, we readministered the conditioning stimulus for 2 minutes. During the second administration of the conditioning stimulus, participants were again instructed to verbally report pain intensity and pain unpleasantness using the same scale as mentioned earlier (Section 2.2.3). Pleasant pain relief was measured immediately after the end of the immersion and every 30 seconds afterwards for 4 minutes. To assess the pleasant pain relief, participants were asked to rate their level of pleasant pain relief on a scale of 0 (“I feel relief, but no pleasure”) to 100 (“I feel relief and the most intense pleasure possible”). These ratings were used to calculate the mean and peak (the highest score) pleasant pain relief of each participant.

## 3. Statistical Analyses

Two paired-sample *t*-tests were conducted. Firstly, we compared pain ratings of the test stimulus before and after the conditioning stimulus, as an index of ICPM efficacy. Secondly, we compared two pleasant pain relief scores, measured after the separate administrations of the conditioning stimulus. To determine the relationship between the conditioning stimulus, ICPM, pleasant pain relief, and subclinical symptoms, Pearson's correlation analyses were performed. We examined potential correlations (i) between pain intensity and pain unpleasantness during the conditioning stimulus and pleasant pain relief (mean and peak), (ii) between ICPM efficacy and pleasant pain relief (mean and peak), (iii) between pain intensity and unpleasantness during the conditioning stimulus and ICPM efficacy, (iv) between psychological symptoms (STAI-S, BDI-II, and SHPS) and pleasant pain relief (mean and peak), and finally (v) between psychological symptoms (STAI-S, BDI-II, and SHPS) and pain (intensity and unpleasantness). The interclass correlation coefficient (ICC) estimate along with the 95% confidence intervals (CI) was calculated for mean pain intensity scores taken during each conditioning stimulus, mean pain unpleasantness scores taken during each conditioning stimulus, and for pleasant pain relief (first pleasant pain relief score taken immediately after each conditioning stimulus). The ICC was calculated using a one-way random effect model, and single measures were reported [41]. This allowed us to determine the test-retest reliability of pain intensity and unpleasantness during both administrations of the conditioning stimulus and of both measures of pleasant pain relief. Values of the ICC that are less than 0.5 are indicative of poor reliability, values between 0.5 and 0.75 are indicative of moderate reliability, and finally, values between 0.75 and 0.90 are indicative of excellent reliability [41]. All variables had a normal distribution, as determined with the Shapiro–Wilk test for normality. All results are presented as mean ± standard error of the mean (SEM) and are considered significant at *p* < 0.05. All analyses were performed using SPSS, version 24.

## 4. Results

### 4.1. Inhibitory Conditioned Pain Modulation Paradigm

#### 4.1.1. Heat Pain Threshold and Tolerance

During the pretest, the thermal pain threshold of participants was 42.3°C ± 0.7, the thermal pain tolerance was 47.2°C ± 0.5, and the T50 was 45.9°C ± 0.4.

#### 4.1.2. Tonic Pain Perception

**Figure 1:**
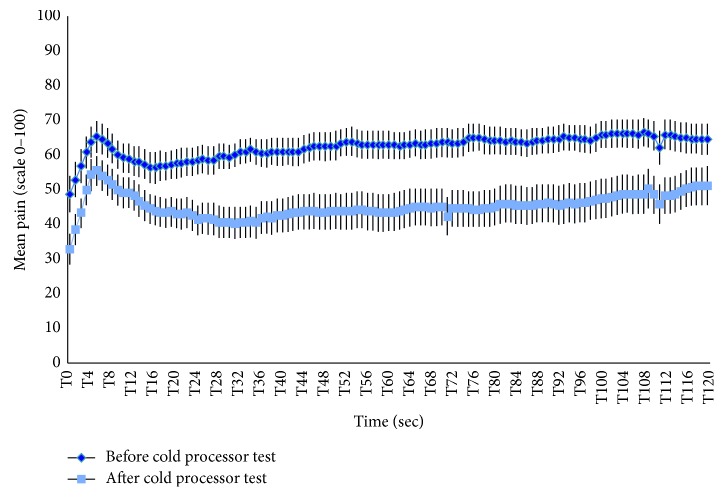
Inhibitory conditioned pain modulation. This figure shows the pain perception of participants during both administrations of the test stimulus for 2 minutes (120 seconds). Pain perception during the test stimulus was evaluated twice, once before (in dark blue) and once after (in pale blue) the administration of the conditioning stimulus. Each time point shows the mean and SEM.

The mean pain ratings for the test stimulus administered before the conditioning stimulus were 67.4 ± 3.3 and were reduced to 51.2 ± 4.7 after the conditioning stimulus (mean difference = 16.1 ± 3.0) (Figure 1). The difference between these pain ratings was significant (*t*(26) = 5.4; *p* < 0.001). During the conditioning stimulus, the mean pain intensity and mean pain unpleasantness were, respectively, 50.9 ± 3.0 and 51.1 ± 4.0.

#### 4.1.3. Pleasant Pain Relief

**Figure 2:**
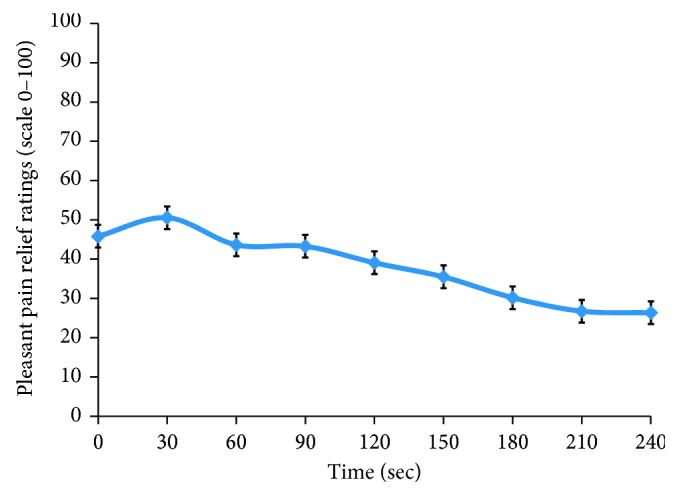
Perception of pleasant pain relief during 240 seconds. This figure illustrates the pleasant pain relief reported by participants for 4 minutes following the second administration of the conditioning stimulus. The mean and SEM are displayed for each time point.

During the second administration of the conditioning stimulus (30 minutes later), the mean pain intensity and mean pain unpleasantness were, respectively, 47.8 ± 3.4 and 47.9 ± 4.0. After this conditioning stimulus, pleasant pain relief measures were taken every 30 seconds for 4 minutes. The mean pleasant pain relief was 40.0 ± 3.8 (Figure 2), and the *peak* pleasant pain relief was 69.3 ± 4.4. It is noteworthy that pleasant pain relief was also measured after the first administration of the conditioning stimulus. No significant difference was found between the two measures (*t*(26) = 0.81; *p*=0.936).

### 4.2. Correlations of Pleasant Pain Relief with Other Psychophysical Measures

**Figure 3:**
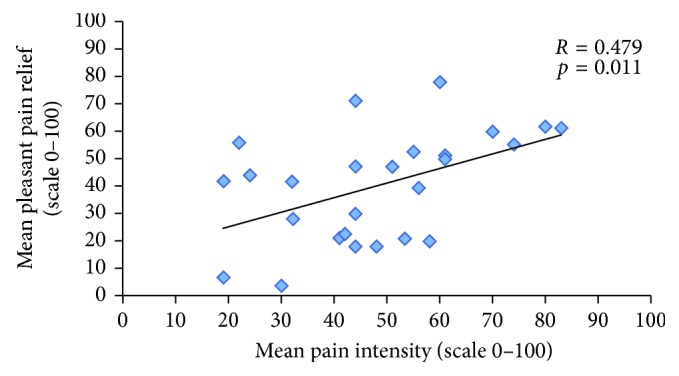
Correlation between pain intensity during the cold-pressor test and mean pleasant pain relief. This figure illustrates the correlation between the mean pain intensity, during the second application of the conditioning stimulus, and the mean pleasant pain relief, measured following the second conditioning stimulus.

**Figure 4:**
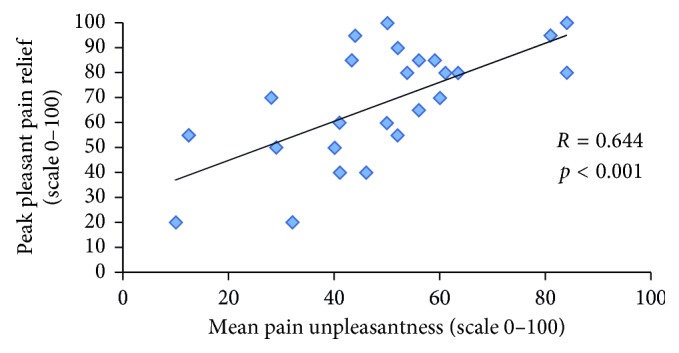
Correlation between pain unpleasantness during the cold-pressor test and peak pleasant pain relief. This figure illustrates the correlation between the mean pain unpleasantness, during the second application of the conditioning stimulus, and peak pleasant pain relief, measured following the second conditioning stimulus.

A significant correlation was observed between *mean* pleasant pain relief and pain intensity during the conditioning stimulus (*r*=0.479; *p*=0.011) (Figure 3). Likewise, a significant correlation was also found between *peak* pleasant pain relief and pain unpleasantness during the conditioning stimulus (*r*=0.644; *p* < 0.001) (Figure 4). Conversely, no significant correlations were found between pleasant pain relief (measured after the first conditioning stimulus) and ICPM efficacy (*r*=0.113; *p*=0.576), as well as between *mean* and *peak* pleasant pain relief (measured after the second conditioning stimulus) and ICPM efficacy (resp., *r*=0.144, *p*=0.47; *r*=0.090, *p*=0.656). Finally, no significant correlations were found between pain intensity during the conditioning stimulus and ICPM efficacy (*r*=0.107; *p*=0.601), as well as between pain unpleasantness during the conditioning stimulus and ICPM efficacy (*r*=0.126; *p*=0.532).

### 4.3. Correlations of Pleasant Pain Relief and Subclinical Psychological Symptoms

Significant correlations were found between mean pleasant pain relief and STAI-S (*r*=−0.402; *p*=0.038). No significant correlations were found between mean pleasant pain relief and BDI-II (*r*=0.814; *p*=0.359) and mean pleasant pain relief and SHPS (*r*=−0.136; *p*=0.498). Finally, no significant correlations were found between BDI-II, STAI-S, and SHPS and pain unpleasantness or pain intensity during the conditioning stimulus (*p* > 0.4).

### 4.4. Test-Retest Reliability

Reliability was evaluated for mean pain intensity and mean pain unpleasantness, taken during two separate administrations of the conditioning stimulus, as well as between each value of pleasant pain relief, taken 10 s after each conditioning stimulus. The ICC correlations along with their 95% CI for mean pain intensity, mean pain unpleasantness, and pleasant pain relief were, respectively, ICC (1,1) = 0.692, 95% CI = 0.434–0.846; ICC (1,1) = 0.870, 95% CI = 0.738–0.939; and ICC (1,1) = 0.638, 95% CI = 0.35–0.816.

## 5. Discussion

The main objective of this study was to examine if there is a relationship between the ICPM efficacy and the pleasant pain relief experienced after the administration of the same conditioning stimulus used to trigger endogenous pain inhibition mechanisms. Associations between pleasant pain relief and other psychophysical measures and subclinical psychological symptoms were also examined. As shown by several previous investigations [5, 7, 42], the conditioning stimulus (e.g., cold-pressor test) produces significant analgesia, as illustrated by a significant reduction in pain perception during the second test stimulus, compared to the first one. Our study showed that significant pleasure was experienced after the interruption of the conditioning stimulus. Greater pain intensity and unpleasantness during the conditioning stimulus was associated with greater pleasant pain relief. However, there was no correlation between ICPM efficacy and the magnitude of pleasant pain relief. Finally, we found that anxiety was negatively correlated with pleasant pain relief.

Prior to analyzing any potential association between ICPM efficacy and the magnitude of pleasant pain relief, it was important to first establish that the interruption of the conditioning stimulus produces significant pleasant pain relief. This was the case. Indeed, in addition to having the mean pleasant pain relief close to 40% and the peak pleasant pain relief close to 70%, the effect also lasted at least 4 minutes in most participants (at endpoint, the pleasant pain relief was 26.3%). By comparison, Leknes et al. [23] measured pleasant pain relief after the interruption of a 15 × 20 mm thermode on the left forehand of the participants during 3 seconds and found that the peak pleasant pain relief was about 35% and lasted about 8 seconds. As in the study from Leknes et al. [23], we found that both pain intensity and unpleasantness during the conditioning stimulus were positively correlated with the magnitude of pleasant pain relief after cessation of the conditioning stimulus. Taken together, these results strengthen the validity of using the cold-pressor test as a conditioning stimulus to elicit pleasant pain relief.

Although the conditioning stimulus elicited strong pleasant pain relief and significant ICPM, pleasant pain relief and ICPM were not significantly correlated. From a methodological point of view, this is an important observation, considering that several teams of investigators use *sequential* ICPM paradigms [28, 43, 44]. A significant positive correlation between the two phenomena would have suggested that the analgesic effects triggered by the conditioning stimulus could be confounded by pleasant pain relief triggered at the end of the conditioning stimulus. The lack of correlation observed here suggests that ICPM assessment is not significantly confounded by the pleasant pain relief effect, although both phenomena co-occur in time.

Another implication of the current study lies in the fact that it provides a new potential explanation for the strong link between pain and anxiety. Although we found no significant relationship in the current study, several previous experimental studies have shown that noxious stimuli cause anxiety, and that anxiety increases pain perception in healthy volunteers [8, 45, 46]. At the moment, however, the reasons for the association between pain and anxiety remain elusive. Despite inconsistent results, some studies have found a negative association between anxiety and the ability to experience pleasure [47, 48]. Comparatively, the link between anxiety and pleasure has been less investigated in experimental settings. Therefore, the finding of a negative correlation between pleasant pain relief and anxiety, as observed in the current study, suggests that anxiety acutely disrupts the homeostatic balance between pleasure and pain. Conversely, a lower ability to experience pleasant pain relief may have caused participants to feel more anxious.

The current study has a few limitations. Firstly, the most prolonged measure of pleasant pain relief (e.g., 240 seconds) was not assessed at the same time as endogenous pain inhibition. However, we found no correlation between pleasant pain relief and ICPM efficacy even when we used the first assessment of pleasant pain relief (e.g., after the first of the conditioning stimulus). This makes it unlikely that the lack of correlation between ICPM efficacy and pleasant pain relief would be confounded by the passage of time. Another limitation of the current study is that the sample size could have been larger, meaning that the lack of correlation between ICPM and pain relief pleasantness could be explained by a lack of statistical power. However, this does not seem very likely given that the correlation between ICPM and pleasant pain relief was very weak. Another limitation has to do with the fact that participants were explicitly introduced to the concept of pleasant pain relief before the experimental session, and this may have influenced participants' expectations of experiencing ICPM. Previous research has shown that the magnitude of ICPM is influenced by expectations [49]. Finally, it is important to remember that the current study used a correlational design, which means that it cannot be concluded from the present results that pleasant pain relief and ICPM are independent phenomena. The experimental manipulation of variables would be required in order to reach a firm conclusion.

## 6. Conclusion

The current study showed, for the first time, that strong feelings of pleasantness are elicited after the cessation of the conditioning stimulus and that ICPM and pleasant pain relief both co-occur but are not significantly correlated. These results provide support for the use of the cold-pressor test as a conditioning stimulus to study pleasant pain relief and suggest that the results of *sequential* ICPM paradigms are not strongly confounded by co-occurring pleasant pain relief. The current results also provide novel insights on the complex link between anxiety and pain perception. Future studies will need to examine the influence of psychophysical properties of nociceptive stimuli (e.g., spatial and temporal summation) on the magnitude of pleasant pain relief and to investigate the neural pathways that are specifically and/or commonly involved in ICPM and pleasant pain relief. Finally, the precise influence of anxiety on pleasant pain relief will need to be determined.

## Figures and Tables

**Table 1:** Characteristics of the participants.

Characteristics	M (%)
Age (M ± SEM)	25.1 ± 0.82

Sex (%)	
Male	40.6
Female	43.8

Ethnicity (%)	
Caucasian	50
Afro-American	6.3
Latin American	3.1
Asian	6.3
Other	18.8

Level of education (%)	
College degree	15.6
Bachelor's degree	40.6
Graduate studies	28.1

Employment status (%)	
Employed	46.9
Unemployed	6.3
Loan or bursary	15.6
Others (i.e., independent worker and welfare)	15.6

Psychological symptoms (M ± SEM)	
BDI-II	5.11 ± 1.07
STAI-S	46.68 ± 0.83
SHPS	48.81 ± 0.65

BDI-II = Beck Depression Inventory; SHPS = Snaith–Hamilton Pleasure Scale; STAI = State and Trait Inventory; SEM = standard error of the mean; M = mean.

## Data Availability

To insure participant privacy, data will not be made available given that genetic data has also been collected as part of this study.

## Abbreviation

ICPM: Inhibitory conditioned pain modulation.
